# Adenoid Disease in School Children

**Published:** 1913-03-15

**Authors:** 


### THE SCHOOL DOCTOR.

### I.—Adenoid Disease in School Children.

One of the most frequently made diagnoses of physical defects in school children is "Adenoids." In the majority of cases it must be admitted that this diagnosis is perfectly correct. Adenoid tissue is a normal constituent of the pharyngeal wall, but it is only when such tissue gives rise to obstruction and to other symptoms and signs that its removal is called for. There is, however, a considerable diversity of opinion among school doctors as to what constitutes the minimum indication for operative interference.

In hospital practice there is already some unanimity. Certain broad lines are laid down, and if the school doctor pays attention to these he will rarely run the risk of having the private family practitioner advising the parent that the adenoids do not need operation. Generally, when adenoids give rise to any one of five well-marked signs and symptoms their removal is indicated. These are: Sepsis, obstruction, deafness or aural trouble, attacks of rheumatism or rheumatic pains, and frequent attacks of tonsillitis. It must be noted that nothing is said here about simple hypertrophy of the tonsils since the indications for the removal of such tonsils are almost the same as for the removal of hypertrophied adenoid tissue in the naso-pharynx. This is a point too often overlooked. An inspection of the mouth is made, and the tonsils are seen to be hypertrophied, in some cases so much so that they appear to meet in the middle line. To obviate mistakes the school doctor should inspect the mouth while the child is perfectly passive, since straining causes the tonsils to bulge forward and may lead to an erroneous diagnosis. For hypertrophy is not sufficient to justify operation; it must be associated with one or other of the indications mentioned above.

The most common indication is undoubtedly sepsis. This is indicated by enlarged cervical glands, especially in the anterior triangles, and frequently by anæmia, foul breath, and what the parent styles "bilious attacks." In so-called "pretubercular" children, removal of adenoids and tonsils often leads to marked improvement of the general health. Obstruction is not so easily discovered as sepsis. Many children are mouth breathers through habit, and not through obstruction. In all cases the medical inspector who examines the child should pay particular attention to the nasal breathing. An ordinary healthy child should be able to breathe comfortably while one nostril is temporarily obstructed by the finger; if the child cannot breathe with one nostril closed there generally exists some obstruction, most commonly due to adenoids, although deflected nasal septa and nasal polypi must not be overlooked. Where there is obvious middle-ear disease with deafness and Eustachian catarrh, an operation for the removal of the adenoids is generally advisable as a preliminary to any treatment of the aural discharge. Slight deafness in school children is most generally associated with adenoids; and with the removal of the septic adenoid tissue there is usually improved hearing.

These are obvious indications, and when they are explained to the parents in a simple and lucid manner there is generally no difficulty in getting the children treated. It is more difficult to explain the importance of removing adenoid tissue and hypertrophied tonsils in children who are subject to rheumatic pains or who have had rheumatic fever. Many medical men even refuse to admit that there is a definite relation between enlarged tonsils and rheumatic fever, but it is a striking fact that if such children have their tonsils and adenoids removed there is almost invariably an amelioration of their general health. Still less generally is it accepted that there is a definite association between chronic appendicitis and hypertrophied adenoid and tonsillar tissue; but that such an association exists few who have studied the matter can have any doubt. In these cases all that the school doctor can do is to point out that the general health is suffering and that the tonsils may, and very likely do, have something to do with this state of ill-health, and that their removal is, therefore, warranted.

Parents sometimes inquire if the operation is likely to be followed by any untoward results. It is laid down as a rule that the school doctor is not to give advice as to treatment, but such direct inquiries cannot be shirked. In general, it must be pointed out that every operation, especially when a general anæsthetic is given, is attended with a certain amount of danger. The operation for the removal of adenoids and tonsils is, however, very often successfully performed without either local or general anæsthesia being used, and this danger, therefore, can be avoided. When the operation is done by a skilled surgeon, the after-effects are almost *nil* so far as difficulties are concerned. In a few cases enucleation may lead to considerable scarring of the palate, and in some children the simple operation of tonsillectomy may produce a temporary change in the voice. This should be pointed out to the parents whose children are choir-boys. In the majority of cases, however, the school doctor may safely say that the operation is devoid of serious risks. For he would not advise such interference if there existed any grave condition—such as hæmophilia, for instance—which augmented the risk. The safe method of procedure is to advise operation only in such children as really need it. To advise it in every case in which a child is a mouth breather, or in which the tonsils are enlarged, is to court indifference and neglect of all subsequent advice, since the parents in many cases prefer to abide by the opinion of the family doctor, who rightly adopts a conservative policy with regard to tonsils and adenoids.

